# Association between gene methylation and experiences of historical trauma in Alaska Native peoples

**DOI:** 10.1186/s12939-023-01967-7

**Published:** 2023-09-08

**Authors:** Mary P. Rogers-LaVanne, Alyssa C. Bader, Alida de Flamingh, Sana Saboowala, Chuck Smythe, Bernadine Atchison, Nathan Moulton, Amelia Wilson, Derek E. Wildman, Alan Boraas, Monica Uddin, Rosita Worl, Ripan S. Malhi

**Affiliations:** 1 Carl R Woese Institute for Genomic Biology, University of Illinois at Urbana-Champaign, Urbana, IL 61801 USA; 2 Department of Anthropology, McGill University, Montreal, QC H3A 2T7 Canada; 3 Sealaska Heritage Institute, Juneau, AK 99801 USA; 4 Program in Ecology, Evolution and Conservation Biology, University of Illinois at Urbana-Champaign (UIUC), Urbana, IL 61801 USA; 5 Kenaitze Indian Tribe, Kenai, AK 99611 USA; 6 Hoonah Indian Association Hoonah, Hoonah, AK 99829 USA; 7 Huna Heritage Foundation, Hoonah, AK 99829 USA; 8 Genomics Program, College of Public Health, University of South Florida, Tampa, FL 33612 USA; 9 Department of Anthropology, Kenai Peninsula College, Soldotna, AK 99669 USA; 10 Department of Anthropology, University of Illinois at Urbana-Champaign, Urbana, IL 61801 USA

**Keywords:** Alaska Native peoples, Community engaged research, Historical trauma, Epigenetics, DNA methylation

## Abstract

**Background:**

Historical trauma experienced by Indigenous peoples of North America is correlated with health disparities and is hypothesized to be associated with DNA methylation. Massive group traumas such as genocide, loss of land and foodways, and forced conversion to Western lifeways may be embodied and affect individuals, families, communities, cultures, and health. This study approaches research with Alaska Native people using a community-engaged approach designed to create mutually-beneficial partnerships, including intentional relationship development, capacity building, and sample and data care.

**Methods:**

A total of 117 Alaska Native individuals from two regions of Alaska joined the research study. Participants completed surveys on cultural identification, historical trauma (historical loss scale and historical loss associated symptoms scale), and general wellbeing. Participants provided a blood sample which was used to assess DNA methylation with the Illumina Infinium MethylationEPIC array.

**Results:**

We report an association between historical loss associated symptoms and DNA methylation at five CpG sites, evidencing the embodiment of historical trauma. We further report an association between cultural identification and general wellbeing, complementing evidence from oral narratives and additional studies that multiple aspects of cultural connection may buffer the effects of and/or aid in the healing process from historical trauma.

**Conclusion:**

A community-engaged approach emphasizes balanced partnerships between communities and researchers. Here, this approach helps better understand embodiment of historical trauma in Alaska Native peoples. This analysis reveals links between the historical trauma response and DNA methylation. Indigenous communities have been stigmatized for public health issues instead caused by systemic inequalities, social disparities, and discrimination, and we argue that the social determinants of health model in Alaska Native peoples must include the vast impact of historical trauma and ongoing colonial violence.

**Supplementary Information:**

The online version contains supplementary material available at 10.1186/s12939-023-01967-7.

## Background

The experiences of Indigenous peoples of North America since European contact include historical and ongoing traumas such as genocide, loss of land and foodways, and forced conversion to Western lifeways, affecting individuals, families, communities, cultures, and health [1–5]. Historical trauma experienced by Native American and Alaska Native peoples is associated with physiological embodiment and has been hypothesized to be associated with epigenetic traits like DNA methylation (e.g. [6–8]). Further, Indigenous cultural connections, community, and revitalization are evidenced as a source of resiliency and healing from trauma [9–11]. To address these complexities, a community-originated research collaboration was established between Alaska Native communities in two regions and researchers at multiple institutions in the United States in order to better understand the relationships between historical trauma and DNA methylation, as well as the cultural connection with wellbeing. We discuss the project’s approach to community engagement, identify associations between the response to historical trauma and DNA methylation, and discuss the impacts of culture on wellbeing in Alaska Native peoples.

Historical trauma is defined as the “cumulative emotional and psychological wounding across generations, including the lifespan, which emanates from massive group trauma” [12]. Notorious examples of historical trauma in Native American and Alaska Native peoples include genocide and cultural genocide, forced relocation, and the boarding school era [4, 5, 11–13]. For example, the Angoon Bombardment, Kake Bombardment, and Wrangell Bombardment in the late 1800s destroyed three Southeastern Alaska villages and their outlying camps [14–16]. Tribal members discussed the significant impacts of boarding schools [5, 17]. Others discussed violence against women, including sterilization without informed consent in the Indian Health Service (1960s—1970s) and high rates of missing and murdered Alaska Native women [18, 19].

Local historical traumas in the regions of Alaska where this research was conducted also included discriminatory policies, such as school policies with harsh punishments barring students from speaking their languages and federal policies barring Alaskan Native people from gathering traditional food sources. For example, the National Park Service prohibited harvesting gull eggs in Glacier Bay (1960s—2014). Traditional ecological knowledge is sustained and nurtured through direct connections with the land, and this long-term disruption created imbalance; when re-legalized, climate change had likely shifted gull egg development and research participants discussed the need to redefine the right timing for egg harvesting. An additional local historical trauma includes a major fire (1940s) that destroyed several clan houses, with subsequent federal funding that banned the rebuilding of clan houses. Further, Wark (2014) provides a thorough ethnotoxicology, ethnohistory, and autoethnography of the local colonial histories of alcohol, arguing against a biological reductionist and for a culturally-informed approach to understanding use of alcohol [20]. Public health disparities in Alaska Native peoples are a source of stigmatization and discrimination, but many of these disparities have been recognized as stemming from the pervasive impact of historical trauma and ongoing colonial violence [21].

Both historically and recently, scientists also committed unethical, discriminatory acts against Indigenous communities, perpetuating colonial violence (e.g. [22, 23]). Given the history of extractive science (also known as helicopter or vampire research, e.g. research not being designed to benefit tribes, researchers failing to engage with tribes, researchers "flying in" to take samples and "flying out" without or rarely returning, and/or publishing or disseminating research findings in ways that actively harm tribes), research with Indigenous peoples must instead be designed to uphold tribal sovereignty [23, 24]. The tribe must be central project decision makers, and researchers should work to develop cultural competency to respectfully engage with their Indigenous research partners, invest in building tribal research capacity, and disseminate research results in collaboration with tribal leadership [24]. In the current project, the communities and research team used practices and values drawn from the Summer internship for INdigenous peoples in Genomics (SING) and models of community-based research that include equitable collaboration, iterative processes, and long-term commitment [25]. Mutual trust and respect for tribal sovereignty are central to our research values.

Here we present a community-engaged approach taken to better understand the relationship between historical trauma and biological embodiment and identify some of the impacts of social support through cultural engagement. We specifically investigated the relationship between historical loss associated symptoms and DNA methylation, measured in whole blood, across the genome. We hypothesized that historical loss associated symptoms are associated with DNA methylation. This hypothesis is grounded in prior work linking historical trauma with social, cultural, and biological changes, furthering the evidence for embodiment of historical trauma [1, 2]. We additionally examined the relationship between cultural identification with general wellbeing. We hypothesized that cultural identification would be positively associated with general wellbeing. This hypothesis is grounded in discussions with community partners, oral narratives, and prior research that highlight the ways interconnected sources of individual, collective, and cultural resilience together can facilitate healing and wellbeing [9].

## Methods

### Study background and participants

One of the Alaska Native tribes critical to this project had initiated a collaboration with the research team to conduct a separate genomics study. Discussions on future collaborations started, and a project on the relationship between historical trauma and epigenetics was suggested by the local healthcare center director, an Indigenous medical doctor from a tribe in the Southwest of North America. The project developed from these conversations and was initiated in that local community in the Kenai Peninsula. The project expanded to include a second region of Alaska (Southeast Alaska) with tribal members living in two locations. In this second region, the project PI (RSM) reached out to local contacts with whom he has previous working relationships. The community already had projects geared towards understanding and addressing trauma, and thus they were also interested in joining the current research study.

Recruiting methods were mainly conducted by tribal members working in the tribal offices and staff working in local Indigenous institutions. Flyers and press releases were also used to advertise the study. Recruiting for the study occurred between 2018–2019. All participants were at least eighteen years of age. All study procedures were approved by tribal review in the two regions and the University of Illinois at Urbana-Champaign Institutional Review Board (UIUC IRB #10538). All participants provided written informed consent. This research was carried out in accordance with the Office for Protection of Research Subjects at the University of Illinois. This office follows regulations at 45CFR 46 of the U.S. Department of Health and Human Services.

To respect sovereignty over data, each individual participant retained the right to withdraw their participation at any time. Further, all samples will be destroyed upon the conclusion of the study to prevent any use beyond what was included in the informed consent process. The methylation data generated in this project will be retained, but it will not be deposited in a public database where it could be used for purposes beyond the intended study. Rather, should a secondary research team want to ask follow-up questions, they will need to work directly with tribal leadership, and project data access for secondary research questions must be covered under the original informed consent documents.

In total, 117 participants joined the research study. Participation in the research study consisted of a survey and blood draw. All aspects of the project were voluntary, and thus some participants skipped questions in the surveys. Recruitment was conducted over multiple research trips where three research trips were made to the Kenai region and two recruiting trips were made to Southeast Alaska. In the Kenai Peninsula, 29 tribal members joined the research project. Of these, two individuals chose to skip the survey portion of the study. In Southeast Alaska, the study included tribal members living in two locations where respectively 64 and 24 individuals joined the research project. Related individuals were removed in some analyses for a total of 96 participants and this decision is specified for each analysis described below.

Participants were asked to complete written research surveys, and participants were given a choice on whether they wanted to answer the questions themselves or as an interview-style with a member of the research team. A member of the research team was always close-by to answer any clarifying questions. Additionally, many of the survey questions were quite personal, could be retraumatizing, or could be triggering. Thus, a counselor was always on hand for participants to choose to talk to before starting the survey, while taking the survey, or after the conclusion of the study. In the Kenai Peninsula, the research study was conducted at a holistic health facility on tribal lands, designed with local architectural details reflecting tribal history and culture. In the Southeast, the project was held at an Indigenous facility, local church/community space, and Southeast Alaska Regional Health Consortium Health Center. In the Southeast, branches from s’áxt (*Oplopanax horridus*, devil’s club) were hung above the door at the recommendation of Alaska Native employees at Sealaska Heritage Institute (SHI). S’áxt is a sacred plant with many traditional spiritual and medicinal properties.

### Surveys

The surveys included questionnaires describing demographic information (including age, sex, gender, and smoking), cultural identification [26], participant’s general wellbeing over the past month [27], and historical loss and historical loss associated symptoms [28]. Missing data was excluded, and all tables include sample size.

Cultural identification consisted of four questions including, “To what degree do you participate in Alaska Native culture?”, “How much of your family lives by Alaska Native culture?”, “How much do you live by Alaska Native culture?”, and “How many people living around you identify as Alaska Native?” [26]. Response options were a 4 Likert scale with response options: a lot, a moderate amount, a little, or none. The scores were reversed so a high score indicates a strong sense of personal identification in Alaska Native culture.

General wellbeing was assessed using a scale that asks 18 questions about how one has been feeling over the past month [27]. The scores from all questions are added together (including a reverse score for 8 items), 14 is subtracted from the total, and the resulting score ranges from 0–110. Scores from 0–60 are categorized as experiencing “severe distress,” scores from 61–72 are categorized as experiencing “moderate distress,” and scores from 73–110 are categorized as experiencing “positive wellbeing” [27].

Participants completed the historical loss and historical loss associated symptoms scales relating to historical trauma in Indigenous peoples of North America [28]. The historical loss scale reflects the frequency of thoughts on historical trauma, and the historical loss associated symptoms scale reflects symptoms experienced when thinking about historical trauma [28]. The ways historical trauma is used and studied encompasses multiple processes; Walters et al. (2011) suggest these historical loss scales measure “historical trauma-related factors (e.g., collective loss) that interact with proximal stressors” [21]. A higher score indicated respectively more frequent reflections on or experiencing more symptoms related to historical trauma. There is not currently a gold standard for scoring these historical loss scales [29]. However, the historical traumas and symptoms on the survey may be locally variable, and we used a previously described scoring method to address this whereby survey responses were first restricted to experiences and symptoms reported by at least the median of each sample and are then summed [30].

### DNA methylation measurements

The blood draw was conducted by phlebotomists employed locally at tribal and regional medical centers. Whole blood samples were collected into EDTA and PAXgene tubes. The EDTA tubes were gently inverted 8–10 times and subsequently frozen. Between one and twelve days after the initial blood draw, samples were shipped on ice overnight from Alaska to the University of Illinois, Urbana-Champaign. DNA was extracted from the EDTA tubes in UIUC’s Malhi Molecular Anthropology Lab using the QIAGEN QIAamp DNA Blood Mini Kits and standard protocol.

DNA methylation was analyzed using the Illumina Infinium MethylationEPIC array. DNA extracts (250 ng) were submitted to the University of Illinois Functional Genomics Unit for processing. Samples underwent bisulfite treatment using the Zymo EZ DNA Methylation-Gold kit and standard protocol. Samples were submitted on two dates and run on 16 arrays. Array sample loading was balanced for historical trauma symptom scores, sex, age, and PTSD. Three control samples were submitted randomized across plates and plate locations, with the primary control sample run six times. Some participants were genetically related, and thus, related individuals were excluded such that one member per family was included in methylation analyses for a final sample size of 96 individuals.

The raw idat files were read into R Studio Version 1.2.5033 using *minfi* for preprocessing and quality control [31–33]. Distributions of methylation Beta-values and M-values for each sample and probe type (I and II) were first visually assessed using *shinyMethyl* [33]. Blood cell type composition was estimated using *FlowSorted.Blood.EPIC*, and estimates are reported in Supplemental Table 3 [34, 35]. Samples were corrected for background noise and dye-bias normalization was performed using normal-exponential out-of-band (Noob) method [36]. Probe intensity not significantly above background signal at *p* > 0.01 were considered failed and removed from analysis. Probe type (I and II) bias was adjusted for using the regression on correlated probes (RCP) method [37]. Potentially cross hybridizing probes (McCartney et al. 2016), probes near common SNPs, and probes located on X and Y chromosomes were removed using *DMRcate* [38, 39]. Finally, batch effect adjustments were made using ComBat in *sva* to remove effects due to technical variation: run date, array, and position on the array [40]. After preprocessing and quality control, a total of 782,841 CpG sites remained in the analysis.

Smoking is associated with changes in gene methylation, so a methylation smoking score was computed to include smoking as a covariate in the EWAS. The smoking score was computed for each individual using the method described in Logue et al. (2020) and effect size estimates for loci associated with smoking pack years reported in Li et al. (2018) [41, 42]. In this sample, the mean smoking scores were significantly different for those who had never smoked (*n* = 13, mean: -37.073, SD: 6.29), had ever smoked (*n* = 61, mean: -30.99, SD: 2.91), and smoked within the past 30 days (*n* = 37, mean: -10.90, SD: 3.73) (one-way ANOVA, *p* < 0.0001).

### Statistical analyses

A multiple linear regression model was created to assess the relationship between cultural identification and wellbeing. The model controlled for participant sample location, age, and sex. Alpha was set at 0.05. All analyses were conducted using R Studio Version 1.2.5033.

An epigenome-wide association study was run using CpGassoc to identify CpG sites associated with historical trauma symptoms. We used a median-split to classify historical trauma symptom scores in “more than median” symptoms experienced and “less than median” symptoms experienced. Model covariates included: cell types (CD8 T cells, CD4 T cells, NK cells, B cells, Monocytes, and Neutrophils), smoking score, sex, and age. Sites were considered significant at the Bonferroni–Holm corrected p-value for multiple comparison (*p* < 6 × 10–8).

Due to sample size, an additional restricted association study was run in *CpGassoc* to identify CpG sites associated with historical trauma symptoms in sites in the glucocorticoid receptor regulatory network (GRRN). Differences in methylation often have small effect sizes, and restricting the analysis to sites located in or near GRRN pathway genes helps identify sites with potential functional relevance [43]. Sites were considered significant at the Bonferroni–Holm corrected *p*-value for multiple comparison (*p* < 1 × 10–5).

The target gene names from the complete GENECODE v12 build for all significant CpG sites were pulled from the Illumina manifest Infinium MethylationEPIC v1.0 B4. Gene names were then uploaded to Reactome to identify potential biological pathways represented in significant results [44].

Finally, methylation may be tissue specific, and thus site methylation associated with historical trauma symptoms in blood samples may or may not be replicated in other tissues. The Blood–Brain Epigenetic Concordance (BECon) tool was used to report correlations between methylation in blood and brain for the top significant CpG sites [45].

## Results

### General survey responses by the study sample

Participants (*n* = 117) joined the research project from two regions of Alaska: the Kenai Peninsula and the Southeast. Related individuals were removed for epigenomic analyses, resulting in 96 participants. Aggregated response data for each of these scales is found in Tables 1, 2 and 3 and Supplemental Tables 1–2. Participant ages ranged from 18 – 85 years (mean 51.26, SD 17.42).

**Table 1:** Summarized survey responses

Survey	Mean	Standard Deviation	Range	n
Sample Location				
*Kenai Peninsula*				29
*Southeast (urban)*				64
*Southeast (rural)*				24
Age	51.26	17.42	18—85	111
Sex				
*Female*				77
*Male*				33
*Other (e.g. Two-Spirt)*				4
Cultural Identification	12.76	2.32	4—16	106
Wellbeing	65.02	18.17	19—103	114
Historical Loss Scale	22.85	6.86	7—36	95
Historical Loss Associated Symptoms Scale	14.88	5.09	6—26	95

Summarized responses for metrics included in this study. This study was conducted in two regions of Alaska (Kenai Peninsula and the Southeast)

**Table 2:** Historical loss scale aggregated survey responses

	**Kenai Peninsula**		**Southeast**	
**Variable**	**Mean**	**SD**	**Mean**	**SD**
Overall Score	36.15	14.08	39.16	13.59
Score for questions endorsed above sample median	23.96	7.66	22.51	6.62
1. The loss of our land	2.46	1.27	2.88	1.41
2. The loss of our language*	3.20	1.51	3.80	1.41
3. Losing our traditional spiritual ways*	3.15	1.57	3.51	1.57
4. The loss of our family ties because of boarding schools	2.17	1.38	2.63	1.58
5. The loss of families from the community to government relocation	2.27	1.43	2.33	1.53
6. The loss of self-respect from poor treatment by government officials	2.64	1.37	2.74	1.65
7. The loss of trust in whites from broken treaties	2.44	1.45	2.70	1.56
8. Losing our culture*	3.59	1.39	3.77	1.61
9. The losses from the effects of alcoholism on our people*	4.07	1.36	4.27	1.41
10. Loss of respect by our children and grandchildren for elders*	3.63	1.71	3.69	1.60
11. Loss of our people through early death*	3.44	1.19	3.54	1.47
12. Loss of respect by our children for traditional ways*	3.35	1.38	3.35	1.49

Historical Loss Scale Aggregated Survey Responses. This table provides aggregate responses on the first part of the historical trauma survey (how often one thinks about specific historical traumas). The questions reported by at least a median of either sample are indicated with an asterisk (*)

**Table 3:** Historical loss associated symptoms scale aggregated survey responses

	**Kenai Peninsula**		**Southeast**	
**Variable**	**Mean**	**SD**	**Mean**	**SD**
Overall Score	27.00	9.89	26.80	10.16
Score for questions endorsed above sample median	15.20	4.53	14.77	5.30
1. Sadness or depression*	3.19	1.11	3.13	1.22
2. Anger*	2.82	1.04	2.78	1.26
3. Anxiety or nervousness*	2.28	1.13	2.37	1.20
4. Uncomfortable around white people when you think of these losses*	1.96	1.09	2.13	1.14
5. Shame when you think of these losses*	2.19	1.30	2.23	1.21
6. A loss of concentration*	2.26	1.23	2.10	1.20
7. Feel isolated or distant from other people when you think of these losses*	2.15	1.38	2.14	1.20
8. A loss of sleep*	2.15	1.19	1.94	1.27
9. Rage	1.94	1.10	1.76	0.99
10. Fearful or distrust the intention of white people	2.19	1.24	2.06	1.13
11. Feel like it is happening again*	2.67	1.19	2.38	1.23
12. Feel like avoiding places or people that remind you of these losses	2.21	1.39	2.04	1.32

Historical Loss Associated Symptoms Scale Aggregated Survey Responses. This table provides aggregate responses on the second part of the historical trauma survey (how one feels when thinking about specific historical traumas). The questions reported by at least a median of either sample are indicated with an asterisk

Cultural identification scores ranged from 4 – 16 (mean 12.76, SD 2.32). General wellbeing scores ranged from 19 – 103 (mean 65.02, SD 18.17) [27, 28]. The historical loss scale in this sample ranged from 7 – 36 (mean 22.85, SD 6.86); the possible range was 6 – 36, with a higher score indicating more frequent reflections on specific historical traumas. The historical loss associated symptoms scale in this sample ranged from 6 – 26 (mean 14.88, SD 5.09); the possible range was 6 – 30, with a higher score indicating reporting feeling more symptoms (e.g. always feeling sadness, anger, anxiety) upon reflection of historical traumas.

### Historical loss symptoms are associated with differential DNA methylation in EWAS

**Fig. 1:**
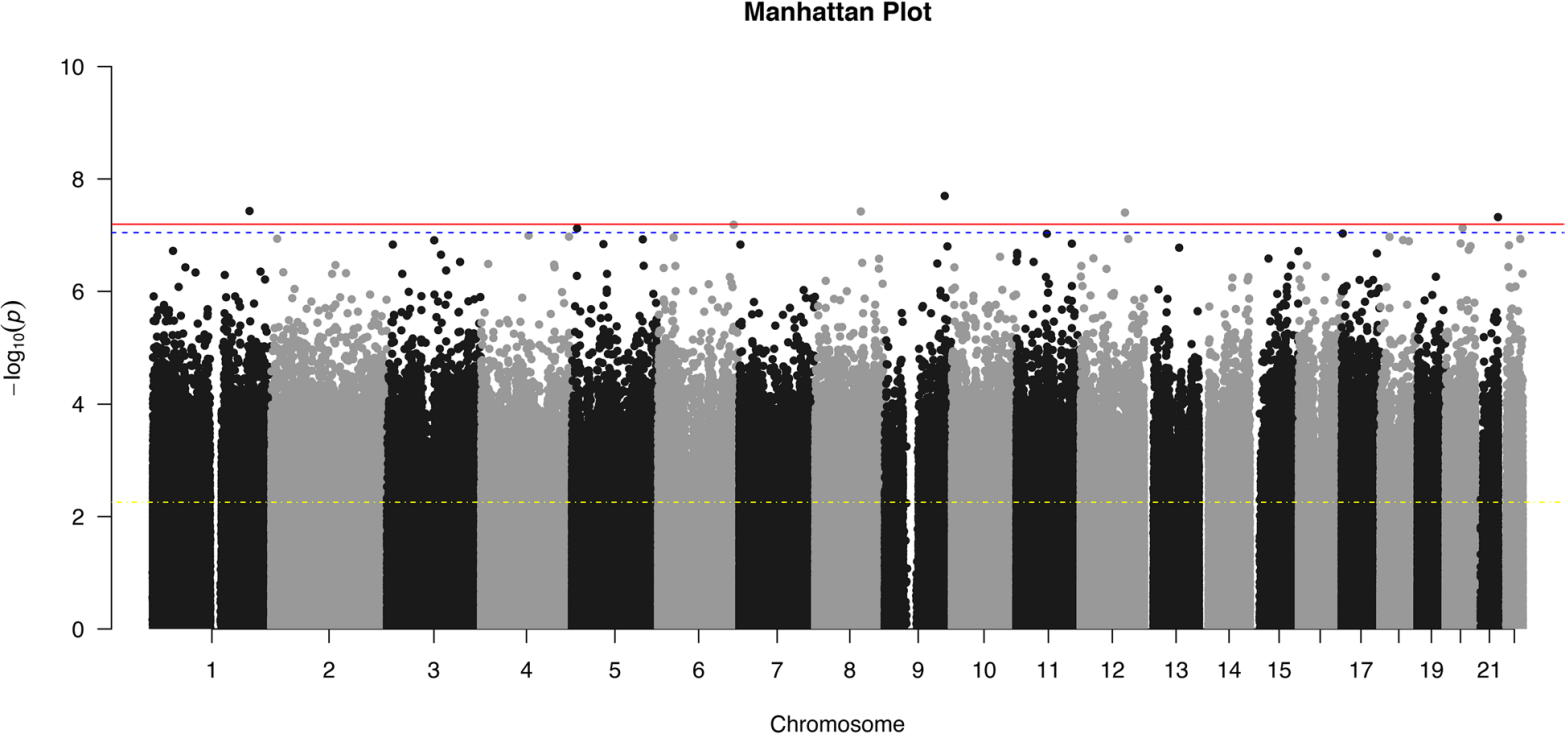
Manhattan Plot displaying scatterplots of –log (*p*-values) for CpG sites across each human chromosome. The top red solid line indicates significance at Bonferroni Correction (Holm method), the dashed blue line just under the red solid line indicates significance at the Mansell et al. (2019) cut-off [46], and the lowest yellow dash-dotted line indicates significance at a False Discovery Rate of 0.05

Five methylation sites were significantly associated with the historical loss associated symptoms scale in the epigenome-wide association study (EWAS) after Bonferroni correction. Figure 1 showcases a Manhattan plot displaying scatterplots of log (*p*-values) for CpG sites across each chromosome.

The top five sites are listed in Supplemental Table 4 including relationship to CpG islands, nearest gene, and regulatory features. All sites are located in OpenSea, and three sites are located within 1500 base pairs of a transcription start site (TSS). The top five CpG sites had an associated target gene listed in the Illumina Manifest from the complete GENECODE build. These genes were: DENN domain containing 1A (*DENND1A*), ATPase plasma membrane Ca2 + transporting 4 (*ATP2B4*), NADH dehydrogenase (ubiquinone) complex I, assembly factor 6 (*C8orf38*), *U6*, and poly(rC) binding protein 3 (*PCBP3*).

**Fig. 2:**
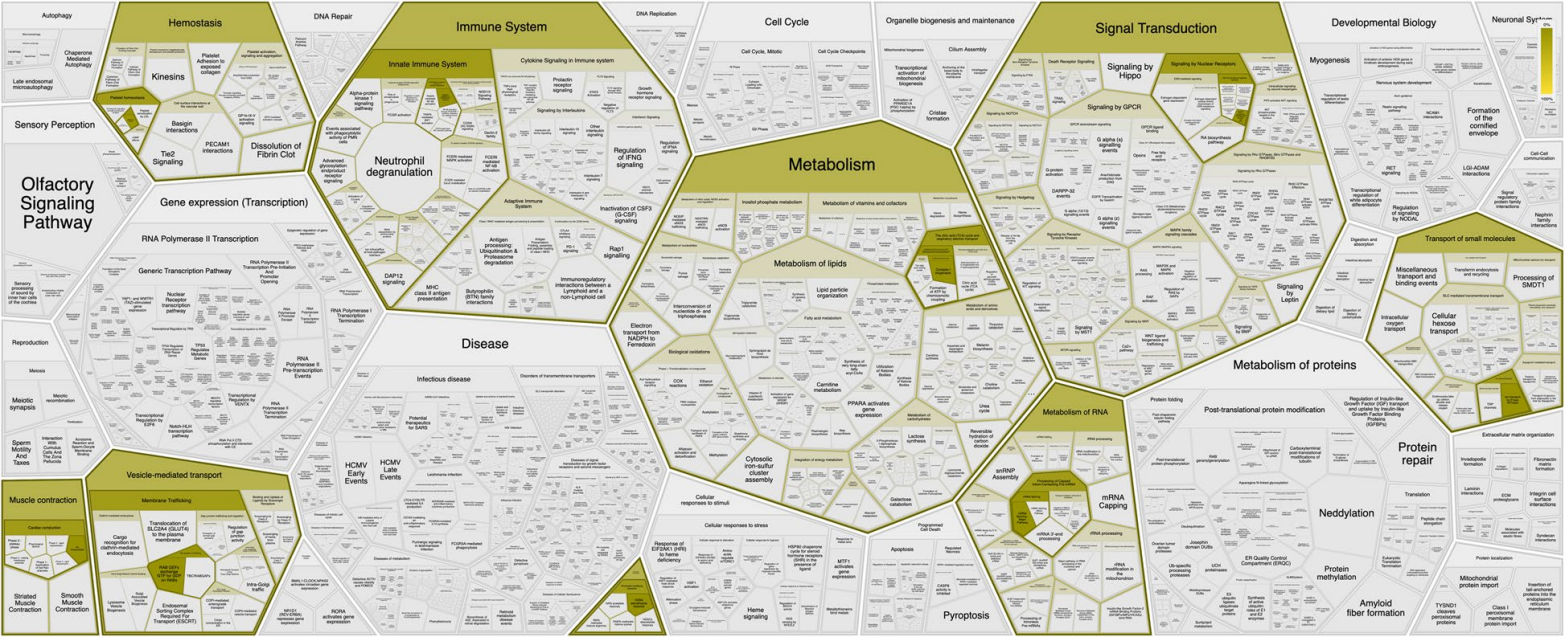
The gene names associated with each significant CpG site were included in a pathway analysis indicating potential biological function using the reactome tool over-representation analysis [44]. The genes were not significantly over-represented in any pathway, but four of the five genes were identified as involved in biological pathways, and this figure highlights the coverage of biological pathways in which these genes play a role. The genes were pleiotropic, meaning each gene likely has a function in more than one biological pathway. The pathways include roles in homeostasis, immune system, metabolism, signal transduction, transport of small molecules, muscle contraction, vesicle-mediated transport, metabolism of RNA, and chromatin organization

While no genes were significantly overrepresented in any biological pathway, the pathway enrichment analysis highlighted multiple pathways these genes were involved with, indicating a pleiotropic nature of these genes. Figure 2 highlights those physiological pathways, which include homeostasis, immune system, metabolism, signal transduction, transport of small molecules, metabolism of RNA, muscle contraction, vesicle-mediated transport, and chromatin organization. Supplemental Table 6 provides additional information on pathways associated with these top five sites. One CpG site at *ATP2B4* had correlation information between blood and brain tissue and was minimally negatively correlated between blood and all brain tissue (*r* = -0.13, supplemental Table 7).

### Historical Loss Symptoms are associated with differential DNA methylation in EWAS restricted to CpG sites in GRRN pathway

An additional restricted EWAS analyses was conducted to identify CpG sites associated with the historical loss associated symptoms scale in genes implicated in the GRRN pathway. Restricting the analysis to sites located in or near the GRRN pathway, a pathway previously implicated in trauma research, may help reveal functionally-relevant sites [43]. Five methylation sites within the GRRN pathway were significantly associated with historical loss associated symptoms after Bonferroni correction. The Manhattan plot and information on associated site locations can all be found in supplemental Fig. 2 and supplemental Table 5. All significant sites had also been previously identified as associated with historical trauma symptoms in the full EWAS within at least the False Discovery Rate (FDR, *p* < 0.05). Four of the five CpG sites are located in OpenSea with one on a north shore of a CpG island. Four of the five CpG sites had an associated target gene listed in the Illumina Manifest from the complete GENECODE build. These genes were: serum/glucocorticoid regulated kinase 1 (*SGK1*), mitogen-activated protein kinase 10 (*MAPK10*), CREB binding protein (*CREBBP*)*,* and heat shock protein 90 alpha family class A member 1 (*HSP90AA1*).

### Cultural identification is positively associated with general wellbeing

**Fig. 3:**
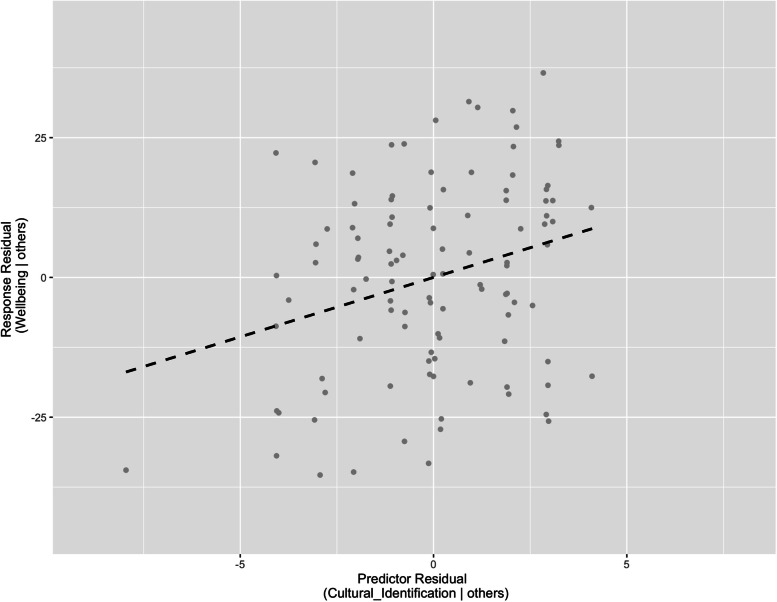
An added variable plot displaying the relationship between cultural identification and general wellbeing (ß = 2.13, *p* = 0.004), while keeping all other variables in the regression model constant (sample location, sex, age). The model formula was: Wellbeing ~ Sample + Sex + Age + Cultural Identification, and the model was significant (*p* = 0.017, *n* = 106, *R*^2^ = 0.15). Those reporting a stronger sense of personal identification in Alaska Native culture tended to have higher general wellbeing scores

There was a positive relationship between cultural identification and general wellbeing (Fig. 3, ß = 2.13, *p* = 0.004; full model *p* = 0.017, *n* = 106, *R*^2^ = 0.15). Increased cultural identification scores indicate strong sense of personal identification in Alaska Native culture, and wellbeing scores above 73 indicate positive wellbeing in the past month.

## Discussion

In this study, a community-engaged research approach was used to better understand relationships between historical trauma and biological embodiment, as well as community identification and wellbeing. The project idea originated from a local Indigenous health care provider, developed in conversation with the local tribal council, expanded to a second region in Alaska with interest in revealing and addressing the effects of historical trauma, and received tribal approvals. Research stage updates with opportunity for feedback were held with leadership at least annually, local tribal members were invited to attend SING, and members of one tribe visited the research lab where samples were housed and analyzed.

Below we discuss the findings that methylation at specific CpG sites was associated with historical loss associated symptoms. We discuss the positive association between cultural identification and general wellbeing. To the best of the authors’ knowledge, this is the first study conducted with Alaska Native peoples to report the connection between the historical loss and DNA methylation, furthering the evidence for embodiment of historical trauma.

### Historical loss symptoms are associated with differential DNA methylation

Methylation at multiple CpG sites is associated with historical loss symptoms. The experience of historical trauma being associated with embodied outcomes has been discussed in oral tradition, personal histories and narratives, as well as academic studies [47]. Phrases including “blood memory,” “soul wound,” “intergenerational trauma,” “the American Indian Holocaust,” and others reflect the many ways historical trauma has been discussed in these multiple spheres to understand the historical trauma experience, impacts, and responses. This study provides an additional type of evidence linking the historical trauma response with DNA methylation.

Embodiment refers to, “how we literally incorporate, biologically, in societal and ecological context, the material and social world in which we live” [48]. This ongoing and complex process is often used in relation to the social determinants of health models such as relating historical trauma and health. The historical loss scales in other Indigenous North American communities have been reported to be associated with anxiety [30], smoking in adolescence [49], and cardiovascular disease risk factors [50]. Similarly, Bombay et al. (2014) reviews evidence that establishes the specific role of Indian Residential Schools as a historical trauma and highlights the relationship between these forced experiences and personal, community, and intergenerational wellbeing [2].

Historical loss symptoms are associated with embodied effects in Indigenous peoples of North America, and the connection between historical trauma and DNA methylation near and/or within genes has been seen in other communities. For example, the experience of historical trauma and DNA methylation was reported by Yehuda et al. (2016), identifying similar methylation patterns in Holocaust survivors and other similar patterns in their descendants [51]. Other types of traumatic experiences and outcomes like post-traumatic stress disorder (PTSD) have also been linked with differential gene methylation patterns in multiple study samples (e.g. [52–54]). Thus, gene methylation is often viewed as a potential mediating link between historical trauma and descendent health disparities. While an EWAS cannot indicate causality or directionality of relationship, linking the historical trauma response with gene methylation provides further evidence of differential gene methylation as another avenue of historical trauma embodiment.

Almost all CpG sites identified are located in OpenSea with associated target genes identified as *DENND1A*, *ATP2B4*, *C8orf38*, *U6*, and *PCBP3* in the full EWAS and *SGK1, MAPK10, CREBBP, and HSP90AA1* in the restricted GRRN EWAS. In relation to other relevant studies, differential methylation in *PCBP3* has been observed in children in a Russian orphanage [55]. Differential expression of *DENND1A* has been observed in patients with major depressive episodes [56]. Differential expression of *ATP2B4* was reported in individuals with PTSD, although differential methylation was not observed [57]. Further, differential methylation in relation to early childhood trauma has been reported in genes with similar functions as those identified in this study, including: guanine nucleotide exchange factors (*DENND1B*, *DENND3*), molecular chaperone (HSP90B1), mitogen-activated protein kinase (*MAPK14*), Poly(RC) Binding Protein (*PCBP2*), and serum/glucocorticoid regulated kinase family member (*SGK3*) [58]. Finally, differential methylation has been reported in *SGK1* and *MAPK1* between individuals with and without PTSD, generalized anxiety disorder, or depression after a traumatic experience; a trend towards differential methylation in *HSP90AA1* was also observed but did not meet the criteria for significance [59]. These studies corroborate relationships between DNA methylation or gene expression in the observed genes with either trauma or responses to trauma.

Pathway analysis reveals that the associated genes are implicated in multiple biological pathways, including signal transduction, metabolism, and immune system. This finding could indicate pleiotropic effects of methylation differences, if methylation at these sites also correlates with changes in gene expression. The pleiotropic nature of genes where methylation is associated with the historical loss symptoms is complemented by evidence of a suite of associations between historical trauma and embodied experiences.

However, as an exploratory study, there are limitations to our understanding of the importance of these findings. First, gene methylation is tissue specific, and thus these findings may not be internally consistent across tissue types. Further, the historical trauma experience ranges widely depending on local history and geographic location, and these results may not be extrapolated to all Indigenous groups. These results use a measure of individual historical loss responses, rather than focusing on the community experience of trauma. There are many ways to measure historical trauma and the historical trauma response [29]; one method may reveal different associations than others. Indeed, historical trauma and the historical trauma response are quite challenging to research as Lehrner et al. (2018) note, “trauma influences multiple levels of the individual’s ecosystem” [60]. Further, we do not distinguish temporally distinct phenomenon as embodiment is an ongoing, multidirectional process [61–63]. Embodiment of historical trauma may be related to other personal, familial, and/or community experiences, and these findings do not control for other experiences of trauma or PTSD. This manuscript does not test intergenerational inheritance – it tests the association between the contemporary experience of historical trauma and one aspect of biological embodiment, gene methylation.

Variation in gene methylation provides one more line of evidence for ways historical trauma interacts with the body. The differences in epigenetic marks complement the multiple ways historical trauma is discussed as having impacted Alaska Native peoples’ culture, health, and communities. The details of these results can help understand the embodiment of trauma in new ways that may be useful to Alaska Native communities as they work to increase resources designed to combat the many effects of historical trauma.

### Cultural identification is positively associated with general wellbeing

There is a positive association between cultural identification and general wellbeing in this sample. Cultural identification reflects the personal sense of identification in Alaska Native culture. We follow Whitbeck et al. (2004), who problematizes the use of acculturation and instead regards enculturation and cultural identification as a source of Indigenous resilience [26]. Cultural connections have been discussed as one of multiple sources of strength and resilience in Native American and Alaska Native peoples [9], and cultural embeddedness has been associated with different aspects of wellbeing in many Indigenous populations (e.g. [64]).

Supporting the link between cultural connection and wellbeing that has been reported in other Indigenous peoples of North America within this sample of Alaska Native peoples may be helpful in understanding or informing approaches to healing from historical trauma. This line of questioning was especially of interest in community discussions about design and interpretation of this project. It also builds on work already being conducted in the regions. For example, Sealaska Heritage Institute (SHI) in Southeast Alaska sponsored a Native arts instruction for incarcerated Native men, with participants overwhelmingly noting that it helped them connect to their culture, helped them develop positive self-identity, and helped a number of the men earn income upon release. SHI also demonstrated how integration of language and culture into programs that serve Alaska Native children supports their emotional health and overall wellbeing [65].

Similar results connecting culture and wellbeing and/or healing from historical trauma are seen in studies in other areas of North America. Brave Heart (1998) describes a psychoeducational group intervention for healing historical trauma in the Lakota, identifying culturally-specific interventions providing relief for participants [13]. In another example, Schultz et al. (2016) analyzes qualitative interviews from six Choctaw women participating in a rewalk of the Trail of Tears, noting that the rewalk transcended Western health approaches to connect participants with traditional Indigenous health knowledge [10]. In each of these examples, different aspects of cultural connection aid in the healing process in ways specific to the participating community.

Drawing from Elm et al. (2016), who conducted qualitative interviews with Two-Spirit Native American and Alaska Native, we do not suggest that cultural identification is the only predictor of wellbeing [9]. Rather, Elm et al. (2016) coined the phrase “braided resilience” to describe the ways Two-Spirit Native American and Alaska Native women pulled from multiple sources to heal from substance abuse and/or mental health challenges [9]. This understanding is more reflective of the ways both communities involved in this study are working to facilitate the healing process in relation to the experience of historical trauma.

Programs directly related to healing from the effects of historical trauma have been initiated in both the Alaska Native communities. For example, in one region, this study was given the name Wooch Yáx Has Kudidáal: Restoring Balance, for calling attention to the embodied effects of historical trauma (Supplemental Fig. 1). In another region, the community built a healthcare building, designed with traditional and historic architectural pieces, using traditional language throughout, and operating with a traditional philosophy of care. The Alaska Federation of Natives, which includes some communities included in the study, passed Resolution 2019–21 on “addressing childhood and historical trauma in our Native communities,” recognizing the impacts since European contact on health and wellbeing and identifying a desire for “comprehensive, integrated, community-wide approaches to successfully achieve healing” [66]. Additionally, both regions have sponsored talks and forums touching on impacts of historical trauma. Both regions have tribal language revitalization programs and projects underway to increase the numbers of speakers and incorporate their language back into the community. The results of this study and previous work on historical trauma in Indigenous communities have the potential to shift approaches to understanding health disparities from one that blames individuals to one that highlights the ongoing impacts of colonial violence [67].

## Conclusion

We conducted a community-engaged research project to better understand embodiment of historical trauma in Alaska Native peoples. This analysis revealed links between historical loss associated symptoms and DNA methylation. Further, there was an association between cultural identification and general wellbeing. Indigenous communities have been stigmatized for public health issues instead caused by systemic inequalities, social disparities, and discrimination, and we argue that the social determinants of health model in Alaska Native peoples must include the vast impact of historical trauma and ongoing colonial violence. In this paper, we also highlight the work the tribes involved in this project have done and continue to do to help address the many impacts of historical trauma in their communities.

### Supplementary Information

**Additional file 1: Supplemental Figure 1.** Wooch Yáx Has Kudidáal Study Poster. **Supplemental Table 1.** Aggregated Survey Responses: Cultural Identification. **Supplemental Table 2.** Aggregated Survey Responses: Wellbeing. **Supplemental Table 3.** Blood Cell Type Estimates. **Supplemental Table 4.** CpG sites associated with historical trauma symptoms. **Supplemental Table 5. **CpG sites associated with historical trauma symptoms in the EWAS restricted to CpG sites in the GRRN pathway. **Supplemental Table 6.** Biological pathways of genes where methylation was associated with historical trauma symptoms. **Supplemental Table 7.** Correlation between methylation in blood and brain at the sites where methylation was associated with historical trauma symptoms. **Supplemental Figure 2.** Manhattan plot for EWAS identifying the association between methylation and historical trauma symptoms for CpG sites at genes in the GRRN pathway.

## Data Availability

The methylation data generated in this project will be retained, but it will not be deposited in a public database where it could be used for purposes beyond the intended study. Rather, should a secondary research team want to ask follow-up questions, they will need to work directly with tribal leadership, and project data access for secondary research questions must be covered under the original informed consent documents.

## Abbreviations

CpG: Cytosine-phosphate-guanine
GRRN: Glucocorticoid receptor regulatory network
TSS: Transcription start site
BECon: The Blood–Brain Epigenetic Concordance tool
FDR: False Discovery Rate
PTSD: Post-traumatic stress disorder
