# Supplementary material for: Association between gene methylation and experiences of historical trauma in Alaska Native peoples
Source: Int J Equity Health. 2023 Sep 8;22:182. doi: 10.1186/s12939-023-01967-7 (PMC10485934; doi:10.1186/s12939-023-01967-7)
Supplement: Supplementary file 1 — Additional file 1: Supplemental Figure 1. Wooch Yáx Has Kudidáal Study Poster. Supplemental Table 1. Aggregated Survey Responses: Cultural Identification. Supplemental Table 2. Aggregated Survey Responses: Wellbeing. Supplemental Table 3. Blood Cell Type Estimates. Supplemental Table 4. CpG sites associated with historical trauma symptoms. Supplemental Table 5. CpG sites associated with historical trauma symptoms in the EWAS restricted to CpG sites in the GRRN pathway. Supplemental Table 6. Biological pathways of genes where methylation was associated with historical trauma symptoms. Supplemental Table 7. Correlation between methylation in blood and brain at the sites where methylation was associated with historical trauma symptoms. Supplemental Figure 2. Manhattan plot for EWAS identifying the association between methylation and historical trauma symptoms for CpG sites at genes in the GRRN pathway. [file 12939_2023_1967_MOESM1_ESM.zip › Supplemental_ Alaska Native Peoples Historical Trauma and Epigenetics Project_20230630.docx]

**Supplementary Information**

Table of Contents

[Supplemental Figure 1 Wooch Yáx Has Kudidáal Study Poster 2](#_Toc139030644)

[Supplemental Table 1: Aggregated Survey Responses: Cultural Identification 3](#_Toc139030645)

[Supplemental Table 2: Aggregated Survey Responses: Wellbeing 4](#_Toc139030646)

[Supplemental Table 3: Blood Cell Type Estimates 5](#_Toc139030647)

[Supplemental Table 4: CpG sites associated with historical trauma symptoms 6](#_Toc139030648)

[Supplemental Table 5: CpG sites associated with historical trauma symptoms in the EWAS restricted to CpG sites in the GRRN pathway 6](#_Toc139030649)

[Supplemental Table 6: Biological pathways of genes where methylation was associated with historical trauma symptoms 7](#_Toc139030650)

[Supplemental Table 7: Correlation between methylation in blood and brain at the sites where methylation was associated with historical trauma symptoms 8](#_Toc139030651)

[Supplemental Figure 2: Manhattan plot for EWAS identifying the association between methylation and historical trauma symptoms for CpG sites at genes in the GRRN pathway. 9](#_Toc139030652)

**Wooch Yáx Has Kudidáal Study Poster**


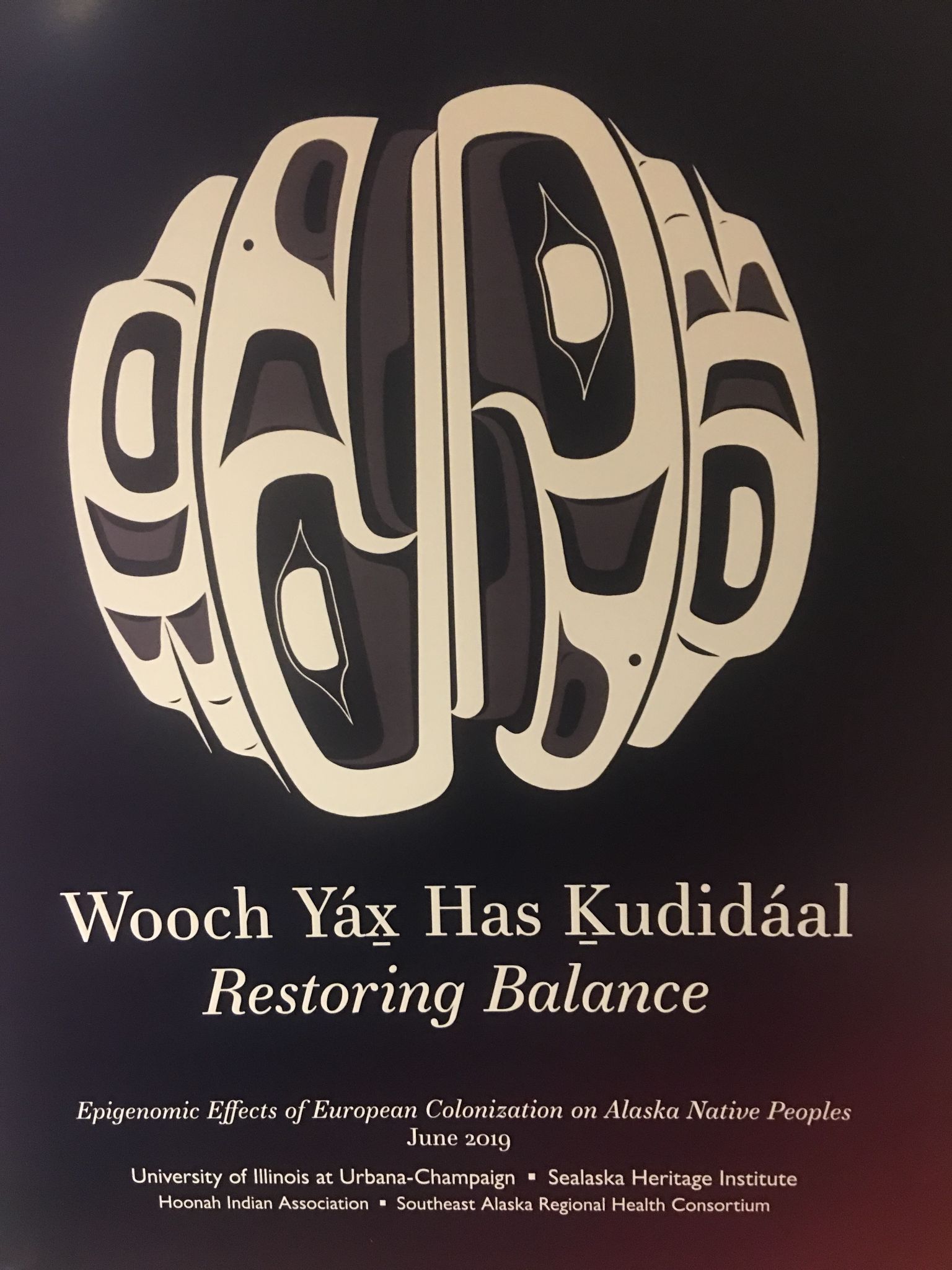


# Supplemental Figure 1 Wooch Yáx Has Kudidáal Study Poster

This figure displays the poster designed for the research study in the Southeast. The name Wooch Yáx Has Kudidáal calls to mind restoring balance. This name is intended to recognize the ways the study calls attention to the embodied effects of historical trauma and point to some ways to address the effects of historical trauma.

**Aggregated Survey Responses: Cultural Identification**

| Variable | Mean | Std. Dev. | Range |
| --- | --- | --- | --- |
| Cultural Identification Total Score | 12.76 | 2.32 | 4-16 |
| Degree participate in American Indian or Alaskan Native culture | 3.12 | 0.81 | 1-4 |
| Family lives by American Indian or Alaskan Native culture | 3.06 | 0.78 | 1-4 |
| Participant lives by American Indian or Alaskan Native culture | 3.03 | 0.81 | 1-4 |
| People living around participant identify as American Indian or Alaskan Native | 3.56 | 0.67 | 1-4 |

# Supplemental Table 1: Aggregated Survey Responses: Cultural Identification

This table provides aggregate responses on cultural identification in Alaska Native peoples living in the Kenai Peninsula and the Southeast.

**Aggregated Survey Responses: Wellbeing**

| Variable | Mean or n | Std. Dev. or % sample | Range |
| --- | --- | --- | --- |
| Wellbeing Total Score | 65.02 | 18.17 | 19-103 |
| Overall Wellbeing |  |  |  |
| ... Moderate distress | 32 | 28.10% |  |
| ... Positive wellbeing | 41 | 36% |  |
| ... Severe distress | 41 | 36% |  |
| Anxiety Subscale | 17.03 | 6.20 | 3-28 |
| Depression Subscale | 9.13 | 4.19 | 1-18 |
| Positive Wellbeing Subscale | 7.53 | 5.11 | 1-17 |
| Self-Control Subscale | 4.34 | 2.64 | 1-13 |
| Vitality Subscale | 12.78 | 4.22 | 3-22 |
| General Health Subscale | 9.22 | 4.16 | 1-16 |

# Supplemental Table 2: Aggregated Survey Responses: Wellbeing

The table provides aggregate responses on current wellbeing.

**Blood Cell Type Estimates**

| Blood Cell Type | Mean | Standard  Deviation | Range | n |
| --- | --- | --- | --- | --- |
| CD8 T | 0.10 | 0.05 | 0.01 - 0.26 | 117 |
| CD4 T | 0.13 | 0.04 | 0.01 - 0.23 | 117 |
| NK | 0.05 | 0.04 | 0 - 0.18 | 117 |
| B cell | 0.05 | 0.02 | 0.01 - 0.16 | 117 |
| Mono | 0.07 | 0.02 | 0.03 - 0.17 | 117 |
| Neu | 0.62 | 0.08 | 0.42 - 0.78 | 117 |

# Supplemental Table 3: Blood Cell Type Estimates

This table presents the composition of cell types in whole blood estimated using the R package FlowSorted.Blood.EPIC (Lucas et al. 2019; Houseman et al. 2012). The cell type estimates were used as a covariate in the final EWAS models as methylation can vary between cell types and cell type composition can vary between individuals.

# Supplemental Table 4: CpG sites associated with historical trauma symptoms

The table is attached in a separate csv. It contains information on the top 5, 8, and 87,134 significant CpG sites where methylation is associated with historical trauma symptoms at Bonferroni cut-off, the p-value cutoff for EWAS studies proposed by Mansell et al. (2019), and False Discovery Rate (FDR). Included in the table are the Illumina MethylationEPIC Manifest CpG labels, EWAS F-statistic, EWAS p-value, EWAS Significance Cutoff (Bonferroni, Mansell, or FDR), and select Illumina MethylationEPIC Manifest information (chromosome, chromosomal position, relation to CpG islands, complete GENECODE target gene names, and complete GENECODE gene region feature category).

# Supplemental Table 5: CpG sites associated with historical trauma symptoms in the EWAS restricted to CpG sites in the GRRN pathway

The table is attached in a separate csv. It contains information on the top 5 and 244 significant CpG sites where methylation is associated with historical trauma symptoms at the Bonferroni cut-off and False Discovery Rate (FDR). Included in the table are the Illumina MethylationEPIC Manifest CpG labels, EWAS F-statistic, EWAS p-value, EWAS Significance Cutoff (Bonferroni or FDR), and select Illumina MethylationEPIC Manifest information (chromosome, chromosomal position, relation to CpG islands, complete GENECODE target gene names, and complete GENECODE gene region feature category).

**Biological pathways involving genes where methylation was associated with historical trauma symptoms**

| **Pathway name** | **#Entities found** | **#Entities total** | **Entities ratio** | **Entities pValue** | **Entities FDR** |
| --- | --- | --- | --- | --- | --- |
| Reduction of cytosolic Ca++ levels | 1 | 12 | 0.00 | 0.01 | 0.09 |
| HDMs demethylate histones | 1 | 26 | 0.00 | 0.01 | 0.09 |
| Platelet calcium homeostasis | 1 | 28 | 0.00 | 0.01 | 0.09 |
| Negative regulators of DDX58/IFIH1 signaling | 1 | 35 | 0.00 | 0.02 | 0.09 |
| NR1H3 & NR1H2 regulate gene expression linked to cholesterol transport and efflux | 1 | 38 | 0.00 | 0.02 | 0.09 |
| NR1H2 and NR1H3-mediated signaling | 1 | 48 | 0.00 | 0.02 | 0.09 |
| Ion homeostasis | 1 | 54 | 0.00 | 0.03 | 0.09 |
| Ion transport by P-type ATPases | 1 | 55 | 0.00 | 0.03 | 0.09 |
| Complex I biogenesis | 1 | 57 | 0.00 | 0.03 | 0.09 |
| DDX58/IFIH1-mediated induction of interferon-alpha/beta | 1 | 81 | 0.01 | 0.04 | 0.09 |
| Platelet homeostasis | 1 | 88 | 0.01 | 0.04 | 0.09 |
| RAB GEFs exchange GTP for GDP on RABs | 1 | 90 | 0.01 | 0.05 | 0.09 |
| Respiratory electron transport | 1 | 103 | 0.01 | 0.05 | 0.09 |
| Rab regulation of trafficking | 1 | 123 | 0.01 | 0.06 | 0.09 |
| Respiratory electron transport, ATP synthesis by chemiosmotic coupling, and heat production by uncoupling proteins. | 1 | 127 | 0.01 | 0.06 | 0.09 |
| Cardiac conduction | 1 | 127 | 0.01 | 0.06 | 0.09 |
| The citric acid (TCA) cycle and respiratory electron transport | 1 | 178 | 0.02 | 0.09 | 0.09 |
| mRNA Splicing - Major Pathway | 1 | 180 | 0.02 | 0.09 | 0.09 |
| Ion channel transport | 1 | 184 | 0.02 | 0.09 | 0.09 |
| mRNA Splicing | 1 | 188 | 0.02 | 0.09 | 0.09 |
| Muscle contraction | 1 | 196 | 0.02 | 0.10 | 0.10 |
| Chromatin organization | 1 | 240 | 0.02 | 0.12 | 0.12 |
| Chromatin modifying enzymes | 1 | 240 | 0.02 | 0.12 | 0.12 |
| Processing of Capped Intron-Containing Pre-mRNA | 1 | 245 | 0.02 | 0.12 | 0.12 |
| Signaling by Nuclear Receptors | 1 | 273 | 0.02 | 0.13 | 0.13 |
| Membrane Trafficking | 1 | 635 | 0.05 | 0.29 | 0.29 |
| Metabolism of RNA | 1 | 675 | 0.06 | 0.30 | 0.30 |
| Hemostasis | 1 | 726 | 0.06 | 0.32 | 0.32 |
| Transport of small molecules | 1 | 731 | 0.06 | 0.32 | 0.32 |
| Vesicle-mediated transport | 1 | 761 | 0.07 | 0.33 | 0.33 |
| Innate Immune System | 1 | 1191 | 0.10 | 0.48 | 0.48 |
| Metabolism | 1 | 2146 | 0.19 | 0.71 | 0.71 |
| Immune System | 1 | 2249 | 0.19 | 0.73 | 0.73 |
| Signal Transduction | 1 | 2574 | 0.22 | 0.78 | 0.78 |

# Supplemental Table 6: Biological pathways of genes where methylation was associated with historical trauma symptoms

Pathway analysis was used to identify pathways involving genes where methylation is associated with historical trauma symptoms. This table displays the pathway names, number of genes within that pathway where there was a site where methylation was associated with historical trauma symptoms, total number of genes within that pathway, and p-value of representation. This table is restricted to the top 5 sites where methylation is associated with historical trauma symptoms after Bonferroni correction.

# Supplemental Table 7: Correlation between methylation in blood and brain at the sites where methylation was associated with historical trauma symptoms

This file is attached as a separate table (Brain-Blood-Correlation_allCpGatFDR_BECon.csv). Some sites where methylation is associated with historical trauma have correlation information between blood and brain tissue. This table lists the one site (at *ATP2B4*) in the top 5 and 8 CpG sites associated with historical trauma, as well as the 30,459 sites (at 10,849 genes) in the 87,134 sites significant at FDR, which have correlation information between blood and brain tissue. The correlations between methylation in blood and all brain tissue range between -0.71 and 0.77.

**Manhattan plot for EWAS identifying the association between methylation and historical trauma symptoms for CpG sites at genes in the GRRN pathway**

# Supplemental Figure 2: Manhattan plot for EWAS identifying the association between methylation and historical trauma symptoms for CpG sites at genes in the GRRN pathway.

This figure displays a Manhattan plot to showcasing log(p-values) for the EWAS testing the association between methylation and historical trauma symptoms at CpG sites at genes in the GRRN pathway. A line is added for Bonferroni and FDR p-value cutoffs, where methylation at 5 sites was associated with historical trauma symptoms at Bonferroni correction and methylation at 244 sites was associated with historical trauma symptoms at FDR.
